# Right-heart infective endocarditis: a propos of 10 cases

**DOI:** 10.11604/pamj.2015.22.280.7441

**Published:** 2015-11-23

**Authors:** Simon Antoine Sarr, Modou Jobe, Malick Bodian, Mbaye Sy, Mouhamadou Bamba Ndiaye, Adama Kane, Alassane Mbaye, Maboury Diao, Moustapha Sarr, Serigne Abdou Ba

**Affiliations:** 1 Service de Cardiologie, CHU Aristide Le Dantec, Dakar, Sénégal; 2 Medical Research Council The Gambia Unit, Fajara, The Gambia; 3 Unité de Formation et de Recherche (UFR), Santé Université Gaston Berger, Saint Louis, Sénégal; 4 Service de Cardiologie, Hôpital Général de Grand Yoff, Dakar, Sénégal

**Keywords:** right heart, infective endocarditis, Dakar

## Abstract

The prevalence and characteristics of right heart endocarditis in Africa are not well known. The aim of this study was to describe the epidemiological, clinical and laboratory profiles of patients with right-heart infective endocarditis. This was a 10-year retrospective study conducted in 2 cardiology departments in Dakar, Senegal. All patients who met the diagnosis of right heart infective endocarditis according to the Duke's criteria were included. We studied the epidemiological, clinical as well as their laboratory profiles. There were 10 cases of right-heart infective endocarditis representing 3.04% of cases of infective endocarditis. There was a valvulopathy in 3 patients, an atrial septal defect in 1 patient, parturiency in 2 patients and the presence of a pacemaker in one patient. Anaemia was present in 9 patients whilst leukocytosis in 6 patients. The port of entry was found to be oral in three cases, ENT in one case and urogenital in two cases. Apart from one patient with vegetations in the tricuspid and pulmonary valves, the rest had localized vegetation only at the tricuspid valve. However, blood culture was positive in only three patients. There was a favorable outcome after antibiotic treatment in 4 patients with others having complications; three cases of renal impairment, two cases of heart failure and one case of pulmonary embolism. There was one mortality. Right heart infective endocarditis is rare but associated with potentially fatal complications.

## Introduction

Infective endocarditis is defined as more or less the extensive damage to the endocardium caused by microorganisms (bacteria or yeast). The microbes are “grafted during bacteraemia on either a healthy or on a previously injured endocardium”. Isolated endocarditis of the tricuspid valve or associated with pulmonary valve defect are less frequent than those of the left.They occur mostly among intravenous drug users, immune-compromised patients and those with congenital heart disease [1, 2]. Their prevalence and characteristics in Africa are not well known.

## Methods

We conducted a 10-year retrospective descriptive multicenter study conducted from January 2000 to December 2010 in two cardiology departments (Aristide Le Dantec University Hospital and Principal Hospital of Dakar) in Dakar, Senegal. We studied the epidemiological, clinical and laboratory profiles of all patients with right-heart infective endocarditis, identified complications, analysed the various prognostic factors of the disease and assessed the management. All patients admitted with infective endocarditis of the right heart according to Duke's criteria: 2 major or 1 major and 3 minor, or 5 minor were included in the study. The data were entered and analysed using Epi Info version 3.5.1. We used Microsoft Excel 2007 for the quantitative data and to calculate averages.

## Results

During the study period, there were 10 cases of right-heart infective endocarditis representing 3.04% of infective endocarditis and 0.055% of all hospitalizations. The average age of our patients was 31 years (range from 9 to 70 years).There was a female predominance with a sex ratio (F:M) of 2.33. The socio-economic profile was low in 3 patients, and was average or good in 7 patients. Three patients had valvulopathies (a mitral stenosis associated with a tricuspid insufficiency, a mitral valve disease associated with a tricuspid insufficiency, an aortic insufficiency associated with a tricuspid insufficiency). A case of atrial septal defect was noted, two parturients and a patient with single chamber pacemaker. One patient reported a fever of long duration. The data of the patients are summarized in Table 1. Clinically 7 patients had a tachycardia with an average heart rate of 127.62 beats/min; nine patients had fever with an average temperature of 38.5^°^C (36.1 to 40^°^C). A tricuspid murmur was found in 9 patients. There were in two patients, a mitral diastolic rumbling; and in one patient an aortic diastolic murmur. There was an interstitial pneumonia and pleural effusion in 3 and 4 cases respectively, four patients had congested hepatomegaly, a case of hemiparesis was noted. The port of entry was found to be oral in three cases, ENT in one case and urogenital in two cases. In one case pacemaker lead was incriminated and in another an iatrogenic cause was found. Laboratory studies found anaemia in 9 cases with a mean hemoglobin of 9.5 g/dl (range from 6.6 g/dl to 11.2 g/dl); leukocytosis in 6 patients. The mean white blood cell count was 21,643.33 /mm3 (6400/mm3 to 44000/mm3).The erythrocyte sedimentation rate in the first hour was increased in four patients, a high CRP level in 8 patients (average of 171.187 mg/l), a hyperfibrino genaemia in 7 patients with a mean of 6.91 g/l. Elevated serum creatinine was observed in 3 patients. 24h proteinuria was found in one case at 1.02 g/24 h and microscopic haematuria with an Addis count in 3 patients with respective values of 222 erythrocytes for the first 2 and 3194 erythrocytes for the third.

**Table 1:** Summary of data of ten patients

	AGE (years)	SEX	CLINICAL FINDINGS	ECHOCARDIOGRAPHY	LABORATORY	EVOLUTION -COMPLICATIONS
1	15	F	Fever TI MVD	Tricuspid vegetations MVD, TI	BC: Negative leukocytosis Hyperfibrinog enaemia CRP: positive	Favorable
2	22	F	HF, TI, AI Pneumonia	Tricuspid vegetations, TI, AI, severe PAH	BC: Negative leukocytosis Hyperfibrinog enaemia CRP: positive	HF Favorable
3	43	F	Fever TI	Tricuspid vegetations, TI	HC: Negative Hyperfibrinog enaemia CRP: positive	Pulmonary embolism
4	27	F	Fever TI Right pleural effusion HF	Tricuspid vegetations TI Seever PAH	BC: Staphylococcus aureus leukocytosis Hyperfibrinog enaemia CRP: positive	HF Favorable
5	22	M	Fever Splitting of S2 Pneumonia,HF	Tricuspid and pulmonary vegetations ASD	BC: Negative Leukocytosis Hyperfibrinog enaemia CRP: positive	HF Favorable
6	39	M	Fever TI Pneumonia	Tricuspid vegetations TI	BC: Negative CRP: negative	Favorable
7	34 years	F	Bilateral pleural effusion TI HF	Tricuspid vegetations Modearte PAH	BC: Negative Leukocytosis Hyperfibrinog enaemia CRP: positive	HF Pulmonary embolism
8	29	F	Fever dyspnoea TI HF	Tricuspid vegetations TI	BC: Negative	HF Favorable
9	09	F	Fever MS, TI Left pleural effusion	Tricuspid vegetations MS, TI	BC: Positive (Streptococcus pneumoniae) Anemia CRP: positive	Favorable
10	70	M	Fever TI Right pleural effusion	Tricuspid vegetations +Vegetations on pacemaker leads, TI	BC: Positive (Pseudomonas aeruginosa) Leucocytosis CRP: positive.	Not favorable, Died of septicaemia.

Blood cultures were performed in 9 patients with an average of 2 sets/patient and were positive in 3 cases. Germs isolated respectively were Streptococcus pneumoniae, Staphylococcus aureus and Pseudomonas aeruginosa. Urinalysis was carried out in 8 patients but was positive in one case. ECG found two cases of a trial fibrillation, a patient with an S1Q3 pattern with background tachycardia. All patients had benefited from a transthoracic Doppler echocardiography and two of whom also had a trans-oesophageal echocardiography. A vegetation or more were found in all patients. In 9 but 1 case, the vegetations were localized on the tricuspid valve, the tricuspid and pulmonary level in one case. Two patients had a large mobile vegetation at the tricuspid valve. In one patient we found a large and numerous vegetations in clusters, located at the pulmonary and tricuspid valves. For the remainder of the patients, we found smaller multiple vegetations. Chest CT scan was performed in one patient in whom pulmonary embolism was suspected which revealed a bilateral pulmonary embolism associated with several areas of pulmonary infarction. Antibiotic therapy was administered in all patients using IV route. Doses were adjusted according to the weight, with renal function taken into account. It was a bi-antibiotherapy in 8 cases involving a gentamicin dose of 3mg/kg/day for an average of 12 days, which was combined respectively with ampicillin in 4 cases, ceftriaxone in four cases, oxacillin in two cases. The outcome was favorable in four patients. However it was associated with complications in others. There were three cases of renal impairment, heart failure in two patients and one case of pulmonary embolism. There was one mortality occurring in a 70-year-old who died from sepsis, after 8 days of hospitalization. The average hospital stay was 39.7 days (8 to 90 days).

## Discussion

Right-heart endocarditis is less common. Its prevalence varies between 5 to 10% depending on the series [1]. Lejkó-Zupanc et al in a paper on 205 patients with endocarditis conducted in 1999, reported 13 cases of right heart endocarditis. It is common to find IV drug users in patients with a right heart endocarditis with the incidence in this population varying from 3 to 5%. In our series, no history of substance abuse was reported. The authors found four patients whose cause was iatrogenic (central venous catheter, pacemaker probe). In four others, there was an immune-compromised status (alcoholic cirrhosis, cancer) [2]. Genital port of entry is not uncommon [3]. Kimbally-kaky also reported 7 cases of endocarditis of the right-heart from illegal abortions in an aseptic environment [4]. We noted a difference in age and sex ratio between our series and those reported in the literature [5]. This is explained by differences in etiologic factors including rheumatic heart disease. Forms of endocarditis in congenital heart disease have been described [6]. Blood cultures are a vital test in the diagnosis of endocarditis. They may however be negative, 14% of cases by Delahaye [7]. The low rate of positive blood cultures found in our series is similar to other series in Africa [4].This finding could be explained by prior antibiotic treatment in most patients with fever, technical conditions in carrying out blood cultures (unsanitary conditions or sample delivery to laboratories), slow growing germs and sometimes a lack of culture plates in our laboratories. Doppler echocardiography plays a major diagnostic role [1]. Germs implicated in a French series were streptococcus in 48% of cases and staphylococcus in 29% [8]. Heart failure may complicate infective endocarditis. It is more common in African series [9] than in the West [10]. One of the most dreaded complications is probably septic pulmonary embolism. Lejkó-Zupanc et al reported in 9 cases out of 13 patients with endocarditis of the right heart. The image most frequently found was that of a pulmonary infiltrate and not that of a pulmonary infarction [1]. Similarly, renal involvement is not uncommon and should be monitored. Its existence, just as advanced age, would be a poor prognostic factor [9]. Mortality in infective endocarditis is still significant between 16-27%, despite the progress in terms of antibiotic therapy [6].

## Conclusion

Right heart infective endocarditis is uncommon. This is a serious condition because of its potentially fatal complications. Circumstances of occurrences and port of entry are variable.
